# Supplementary material for: Naturally Arising de Novo Open Reading Frames as Potential Zinc Chelators in Drosophila melanogaster
Source: Genome Biol Evol. 2026 Oct 30;18(11):evag274. doi: 10.1093/gbe/evag274 (PMC13756084; doi:10.1093/gbe/evag274)
Supplement: Supplementary file 1 [file evag274_supplementary_data.zip › popgen_supplement.pdf]

Supplementary Text

1 Model

Suppose a recurring cellular demand D for Zn^{2+}-buffering capacity is met with the aggregate output of a large pool of N_{pool} independent, non-linked loci that are transcribed and translated. Assuming that overall enzymatic activity is additive with no deleterious, off-target protein-protein interactions, we may define the genome's aggregate Zn^{2+}-buffering capacity C as,

$$C \;=\; \sum_{i=1}^{N_{\text{pool}}} c_i, \tag{1}$$

where i indexes the N_{pool} contributing loci. We may define fitness as a continuous, once differentiable function whose value depends on the difference between the genome's aggregate capacity C and the demand D resulting from existing environmental conditions such that

$$w \;=\; f(C - D). \tag{2}$$

Marginal per-locus selection. Where the genome is well-adapted to local conditions, i.e., the reservoir comfortably meets demand so that $f'(C-D)$ is small (though nonzero), we may consider a single mutation in locus i that alters c_i, and thus C, by Δc_i. Such mutations may include, but are not limited to, slippage events, frameshifts, substitutions, or other heritable activating or disabling events at locus i. To first order, the change in fitness resulting from such mutation is

$$\Delta w_i = f'(C-D)\,\Delta c_i \;+\; O(\Delta c_i^2). \tag{3}$$

Re-writing the leading term of Eq. (3) as the identity

$$\Delta w_i \;\approx\; \underbrace{f'(C-D)\,C}_{\text{(I)}} \;\cdot\; \underbrace{\frac{c_i}{C}}_{\text{(II)}} \;\cdot\; \underbrace{\frac{\Delta c_i}{c_i}}_{\text{(III)}}, \tag{4}$$

we may see that the change in fitness is related to (I) demand saturation, (II) functional redundancy, and (III) the intrinsic effect of mutation i.

2 Per-locus neutrality below the drift barrier

In a genome that is well-adapted to local conditions, and a large number of similarly functioning loci contribute to C, (I) and (II) are small. Specifically, following Hartl–Dykhuizen–Dean [1], such a genome operates where fitness is nearly insensitive to further capacity, $f'(C-D) \approx 0$, so any single locus's marginal change in C barely moves fitness. Similarly, the assumption that all loci function similarly does not allow any single locus to carry much more than its fractional contribution $1/N_{\text{pool}}$, where $1/N_{\text{pool}} \to 0$ for large N_{pool}. As a result, the marginal fitness effect of locus i is small in both structural factors ($f'(C-D)\,C$ by saturation and $1/N_{\text{pool}}$ by redundancy) even when $|\Delta c_i/c_i|$ is of order-of-magnitude 1, as

$$|\Delta w_i| \;\approx\; f'(C-D)\,C \cdot \frac{1}{N_{\text{pool}}} \cdot \left|\frac{\Delta c_i}{c_i}\right|. \tag{5}$$

This relationship demonstrates how a single mutation in locus i is effectively neutral once its coefficient falls below the nearly-neutral drift barrier [2, 3, 4]

$$|\Delta w_i| \;\lesssim\; \frac{1}{2N_e}. \tag{6}$$

Thus, for *D. melanogaster*, with $N_e \approx 1 — 2.5 \times 10^6$ [6, 7], the barrier is

$$\frac{1}{2N_e} \;\approx\; 2 \times 10^{-7} — 5 \times 10^{-7}. \tag{7}$$

Assuming a pool of $N_{\text{pool}} \approx 7{,}000$ *de novo* loci [5], the product $f'(C-D)\,C \cdot |\Delta c_i/c_i|$ would need to exceed $\approx 1.4 \times 10^{-3} — 3.5 \times 10^{-3}$ to clear this barrier. Consequently, even if the entire buffering function carried a modest marginal fitness weight $f'(C-D)\,C$ of order 0.1%, a single locus would need to change its own output $|\Delta c_i/c_i|$ by a full 140% — 350% to be readily selectable in *D. melanogaster*. Since abolishing an entire locus is only a 100% change, no disabling mutation at a single locus clears the barrier.

References

[1] Hartl DL, Dykhuizen DE, Dean AM. 1985. Limits of adaptation: the evolution of selective neutrality. *Genetics* 111:655–674.

[2] Kimura M. 1968. Evolutionary rate at the molecular level. *Nature* 217:624–626.

[3] Ohta T. 1973. Slightly deleterious mutant substitutions in evolution. *Nature* 246:96–98.

[4] Lynch M. 2011. The lower bound to the evolution of mutation rates. *Genome Biol. Evol.* 3:1107–1118. doi:10.1093/gbe/evr066.

[5] Grandchamp A, et al. 2023. Population genomics reveals mechanisms and dynamics of *de novo* expressed open reading frame emergence in *Drosophila melanogaster*. *Genome Res.* 33:872–890 (PMID: 37442576).

[6] Emerson JJ, Cardoso-Moreira M, Borevitz JO, Long M. 2008. Natural selection shapes genome-wide patterns of copy-number polymorphism in *Drosophila melanogaster*. *Science* 320:1629–1631. doi:10.1126/science.1158078.

[7] Kreitman M. 1983. Nucleotide polymorphism at the alcohol dehydrogenase locus of *Drosophila melanogaster*. *Nature* 304:412–417. doi:10.1038/304412a0.
