# Supplementary material for: Naturally Arising de Novo Open Reading Frames as Potential Zinc Chelators in Drosophila melanogaster
Source: Genome Biol Evol. 2026 Oct 30;18(11):evag274. doi: 10.1093/gbe/evag274 (PMC13756084; doi:10.1093/gbe/evag274)
Supplement: Supplementary file 1 [file evag274_supplementary_data.zip › Supplementary Material.docx]

**Supplementary Material**

**Figure S1. Histidine is not particularly enriched or prevalent in neORFs.** Percentage of residues calculated for (A) histidine, cysteine, aspartic acid, glutamic acid, and (B) all amino acids across all gene classes.

**Figure S2. Top 15 most enriched SLiM in neORFs and H-x-H motif prevalence.** Motif density for SLiM (ELM catalog) showing largest difference between neORFs and a length-matched proteome sample. Top row shows H-x-H, which was not present in ELM SLiM.

**Figure S3. Motif and amino acid prevalence by gene class.** Prevalence of (A) H-x-H, (B) C-x-C, and (C) C-x-x-C motifs as well as (D) cysteine and (E) histidine within intergenic, neORFs, young (including duplicate, de novo, and orphan classes), older, and metal-binding gene classes. P-values calculated by (A-C) Fisher’s Exact, or (D-E) binomal test using intergenic genes as a reference.

**Figure S4.** Apo structure prediction does not, on its own, recover calprotectin's metal site or known coordinating geometry for the S100A9 subunit of calprotectin, whose zinc/manganese site is experimentally established.

*‘Dataset_S1_neORF_expression.csv’*

**Dataset S1.** Per-replicate expression (TPM) of the 466 quantified, uniquely mappable, non-overlapping de novo neORFs in S2R+ cells across control, zinc, and manganese conditions and three genotypes, with H-x-H motif content and dm6 coordinates.

| **Strain / species** | **(CA)n units** | **H-x-H** | **Note** |
| --- | --- | --- | --- |
| **Within *D. melanogaster* (strains)** | | | |
| *D. melanogaster* dm6 (ISO1, ref.) | 9 | Y | reference assembly |
| OreR / w1118 (lab) | 9 | Y | identical to reference |
| Denmark (DK) | 6 | Y | 6-bp in-frame deletion (tract shortened) |
| Spain (ES) | 8 | Y‡ | 2- & 1-bp indels (frameshift) |
| Finland (FI) | 6 | Y | 6-bp in-frame deletion (tract shortened) |
| Sweden (SE) | 10 | Y‡ | 2-bp deletion (frameshift) |
| Turkey (TR) | 8 | Y‡ | 2- & 1-bp indels (frameshift) |
| Ukraine (UA) | 9 | Y | identical to reference |
| Zambia (ZI) | 8 | Y‡ | 2- & 1-bp indels (frameshift) |
| **Across *Drosophila* (species)** | | | |
| *D. melanogaster* | 9 | Y | ~0 Mya; reference |
| *D. simulans* | 6 | Y | ~3-5 Mya; strong |
| *D. sechellia* | 7 | Y | ~3-5 Mya; strong |
| *D. mauritiana* | 9 | Y | ~3-5 Mya; strong |
| *D. yakuba* | 7 | Y | ~10-13 Mya; strong |
| *D. erecta* | 5 | Y | ~10-13 Mya; strong |
| *D. ananassae* | 0 | N | ~15-20 Mya; moderate; tract collapsed |
| *D. pseudoobscura* | 5 | N | ~25 Mya; partial; ORF truncated pre-tract |
| *D. virilis* | 0 | N | ~40-50 Mya; fragmentary; ORF truncated pre-tract |

**Table S1.** (CA)n tract length and His-motif status at the ZMEG locus within *D. melanogaster* and across *Drosophila*.  *‡, His motif present but the (CA)n tract is frameshifted (in-frame His run disrupted).*

*‘Dataset_S2.csv’*

**Dataset S2.** Per-protein H-x-H / C-x-x-C cluster counts and codon usage for neORFs, the *Drosophila melanogaster* proteome, and random ORFs.

‘popgen_supplement.pdf’

**Supplementary Text.** A population genetic analysis of the Dykhuizen-Hartl effect using *D. melanogaster* data.
